# Telehealth-Based Family Conferences with Implementation of Shared Decision Making Concepts and Humanistic Communication Approach: A Mixed-Methods Prospective Cohort Study

**DOI:** 10.3390/ijerph182010801

**Published:** 2021-10-14

**Authors:** Tzu-Jung Chou, Yu-Rui Wu, Jaw-Shiun Tsai, Shao-Yi Cheng, Chien-An Yao, Jen-Kuei Peng, Tai-Yuan Chiu, Hsien-Liang Huang

**Affiliations:** 1 Department of Family Medicine, National Taiwan University Hospital, Taipei 100, Taiwan; joycechou0307@gmail.com (T.-J.C.); jawshiun@ntu.edu.tw (J.-S.T.); scheng2140@gmail.com (S.-Y.C.); yao6638@gmail.com (C.-A.Y.); jimmy650228@gmail.com (J.-K.P.); tychiu@ntuh.gov.tw (T.-Y.C.); 2 Department of Family Medicine, Taitung Christian Hospital, Taitung 950, Taiwan; andrewwu09@gmail.com; 3 Department of Family Medicine, National Taiwan University College of Medicine, Taipei 100, Taiwan; 4 New Southbound Health Center, National Taiwan University Hospital, Taipei 100, Taiwan

**Keywords:** COVID-19, clinical encounter, family conference, shared-decision making, telehealth

## Abstract

Smartphone-enabled, telehealth-based family conferences represent an attractive and safe alternative to deliver communication during the COVID-19 pandemic. However, some may fear that the therapeutic relationship might be filtered due to a lack of direct human contact. The study aims to explore whether shared decision-making model combining VALUE (Value family statements, Acknowledge emotions, Listen, Understand the patient as a person, Elicit questions) and PLACE (Prepare with intention, Listen intently and completely, Agree on what matters most, Connect with the patient’s story, Explore emotional cues) framework can help physicians respond empathetically to emotional cues and foster human connectedness in a virtual context. Twenty-five virtual family conferences were conducted in a national medical center in Taiwan. The expression of verbal emotional distress was noted in 20% of patients and 20% of family members, while nonverbal distress was observed in 24% and 28%, respectively. On 10-point Likert scale, the satisfaction score was 8.7 ± 1.5 toward overall communication and 9.0 ± 1.1 on meeting the family’s needs. Adopting SDM concepts with VALUE and PLACE approaches helps physicians foster connectedness in telehealth family conferences. The model has high participant satisfaction scores and may improve healthcare quality among the pandemic.

## 1. Introduction

A family conference is an important therapeutic instrument for physicians to enhance communication and alleviate suffering among patients and family members [1,2]. However, in response to the Coronavirus disease 2019 (COVID-19) pandemic, worldwide public health measures, such as social distancing, quarantine of affected communities, and limitation of population movements, have been proposed to reduce transmission [3,4,5]. Since healthcare facilities could be sources of contagion, hospitals were limiting visits from family and friends. Patients and families are facing distressing restrictions on their caring involvement due to contagious risk [6]. As a result, visitor restrictions have disrupted not only the connection among patients, their families, and health care professionals but also hindered the physician–patient/family face-to-face interaction, including family conferences. Goals-of-care discussions for COVID-19 and other serious illnesses have become more difficult because family members often cannot witness how ill patients have become, and physicians cannot easily communicate with multiple family members at the same time [7,8]. To overcome the ramification of strict visitor limitations, a virtual family conference via telehealth represents an attractive and safe alternative for avoiding person-to-person contact and exposure in a crowded hospital [9].

Family conferences enable health care professionals to share information, clarify doubts, and assess patients’ and families’ needs [2]. With tough decisions, there is an even greater imperative to engage patients and their families regarding care options, creating a unique role for shared decision making (SDM) when conducting family conferences [10]. SDM, as incorporating individual values into best evidence practice to achieve an informed decision, gradually becomes center stage in health care as a cornerstone of person-centered care [11,12,13]. Family members of patients with acute or chronic illness as main caregivers often experience significant psychological distress that needs compassionate professionals to relieve the stress [14]. The existing evidence-based guidelines for family-centered care have suggested that physicians should use structured approaches, such as the “VALUE” approach (Value family statements, Acknowledge emotions, Listen, Understand the patient as a person, and Elicit questions) [15]. This communication strategy was validated by a multicenter randomized trial, in which physicians employed the VALUE approach for family conferences and had significantly reduced symptoms of post-traumatic stress disorder, depression, and anxiety among family members at 90 days after the patients’ death [16]. Furthermore, Zulman et al. also proposed an evidence-based framework—“PLACE” (Prepare with intention, Listen intently and completely, Agree on what matters most, Connect with the patient’s story, Explore emotional cues) to foster physician presence and connection in clinical care that could also be used during the family conference [17]. Active listening, expressions of empathy, and making supportive statements around decision-making can facilitate physician–patient/family relationships [18,19].

The COVID-19 pandemic has resulted in a rapid surge of telehealth in clinical practice to prevent the risk of infection and address healthcare workforce demands [20,21,22]. Many countries are formally relaxing privacy protection regulations and broadening reimbursement policies for telehealth [23,24,25]. As family conferences transition from in-person to virtual context, questions emerge about whether it will impede the meaningful physician–patient/family relationship due to lack of direct human contact during difficult discussions [26,27,28]. Our team had conducted a pilot study using smartphone-enabled telehealth to provide family conferences and effectively reaching a consensus on end-of-life care goals [29]. Nevertheless, virtual physician–family interaction, especially with emotion recognition and response, remains unclear [30,31]. In recognizing the vital role of telehealth during the pandemic, participants’ experiences on communication using telehealth deserve further investigation.

The study aims to investigate whether incorporating the SDM model combining VALUE and PLACE approach in telehealth-based family conferences help health care professionals foster human connectedness and enhance virtual physician presence. We hypothesize that telehealth family conference is an effective modality, and communication skills applicable to face-to-face can also be applied to telehealth. Knowledge of the physician–patient/family interaction and communication pattern in telehealth family conferences are essential to disseminate the model and promote the quality of care for patients, especially in the COVID-19 pandemic.

## 2. Materials and Methods

### 2.1. Framework of Telehealth-Based Family Conference Model with Integration of Shared Decision Making Concepts

**Figure 1:**
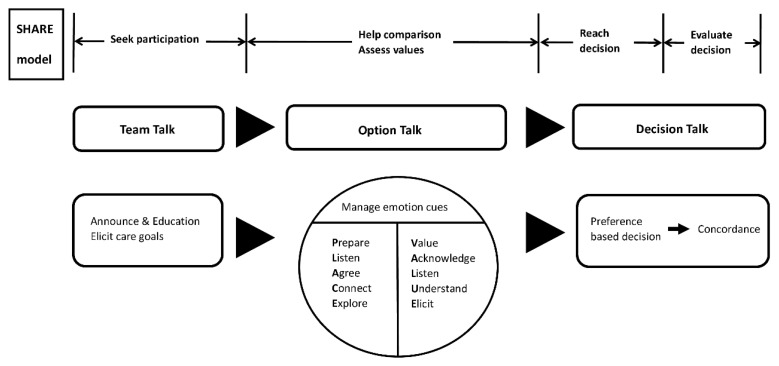
The framework of telehealth-based family conference model integrating shared decision-making concepts with PLACE and VALUE approach.

Family conferences offered a pathway to clarify prognosis and care options, provide psychological support, and facilitate communication. Since the nature of medical care decisions was often preference-sensitive, it was particularly important to pay attention to deliberation and SDM with the patient and family [32]. Therefore, we introduced the SDM model into family conferences. Basic components of SDM were conceptualized in the 3-talk model, including “team talk”, “option talk”, and “decision talk” with the integration of the concepts of SHARE (Seek participation, Help comparison, Assess values, Reach decision, Evaluate decision) proposed by Elwyn et al. and the Agency for Healthcare Research and Quality [12]. The framework of the telehealth family conference is shown in Figure 1.

The “team talk” emphasized seeking patients’ and family’s participation and required participants to prepare the platform for telehealth. The announcement of the family conference date and the education of the medical team, patients, and family about the telehealth software using were essential during the pre-conference phase. At the beginning of the conference, the goal of care, which was the main focus of communication, was also elicited through the “team talk” process.

The “option talk” focused on “help comparison” and “assess values”, which is the main focus of our study. Physicians provided personalized explanations about the risks and benefits of future management. The patients and family often experienced psychological distress alongside the disease trajectory, and emotions such as anger, sadness, or frustration may appear when exploring personal values. Based on a previous study, communication through telehealth allows users to express and interpret emotion in a similar way to face-to-face interaction [33]. Therefore, we assumed that communication strategies that are applicable to face-to-face could be applied to telehealth as well. Researchers had summarized communication strategies for family conferences into a mnemonic for five features: VALUE (value, acknowledge, listen, understand, and elicit) in practical guidance [16,34]. Furthermore, a previous study explored the ways physicians create connection and defined physician presence as three categories of behavior: purposeful intention to connect, conscious navigation of time, and proactive management of technology and environment to focus attention on the patient [35]. A final set of five recommendations with the mnemonic PLACE (prepare, listen, agree, connect, and explore) was proposed. In order to build therapeutic relationships in the virtual environment and smoothen the flow of the family conference, the “VALUE” and “PLACE” approaches were proposed to maintain the physician’s presence and manage the verbal and non-verbal emotion cues during the option talk.

To reach a preference-based decision was the main goal of “decision talk”. The end of the virtual family conference was an opportunity to solidify the connection. Physicians could summarize what had been addressed and the patient and family’s preferences. In this way, physicians conveyed to the family that they listened carefully and wanted to provide care aligned with the patient’s values. Once the medical team reached a consensus with the patient and family on future care plans, the results were documented and uploaded to the electronic medical record system of the hospital.

### 2.2. Study Design and Setting

We conducted a single-center, mixed-methods prospective cohort study from February to May 2020. The study framework was established by the corresponding author and implemented for patients both in the inpatient unit and outpatient department in National Taiwan University Hospital, a tertiary medical center in Taiwan. The hospital set a restriction on family visits and allowed only one caregiver for each patient during the COVID-19 pandemic. Target participants were patients aged 20 years or older in the National Taiwan University Hospital inpatient and outpatient units. Family conferences were conducted if patients or their families required a medical update of prognosis, an open line to discuss the goal of care or a potential discharge plan during the study period. Consenting patients aged ≥20 years were eligible to enroll if they required a telehealth-based family conference. Those who declined to conduct virtual conferences or did not possess the technical skills to participate were excluded. We have conducted a pilot observational study using SDM concepts and VALUE approach in smartphone-enabled telehealth-based family conferences, and the result was highly satisfactory, especially in reaching a consensus on care decisions [29]. Physicians were trained to use the structured and templated SDM framework combining the VALUE and PLACE approach to share information and foster human connectedness in virtual family conferences (Figure 1). Guidelines for conducting family conferences [12,36,37] and original researches on the VALUE [16] and PLACE [17] concepts were provided as supplement education. Group discussions and training seminars were held at the start of the study to reinforce physicians’ practice skills. The family conferences were held by a medical team consisting of physicians specializing in family medicine and palliative care, clinical psychologist, and nurses. The prospective design allowed our study group to follow up on the participants’ experiences over time.

### 2.3. Collection of Family Conference Information and Statistical Analysis

Quantitative and qualitative data of the family conferences were documented by the medical team, including physicians, nurses, and clinical psychologists. The communication goals of the conferences, the attending health care professionals, family members, and the duration of the conference were collected as baseline demographic data. For descriptive findings, data based on categorical variables were presented as percentages, while those based on continuous variables as mean with standard deviation (SD). The records of the communication process in the conference for analysis were under the themes of the VALUE approach, which included two main domains: 1. the patient and family’s emotional cues, and 2. the physician’s response and empathetic statement. The emotion cues from the patient and family were recorded based on consensus after the literature review and experts’ discussion [38]. The emotional state of the patient and family included three categories: verbal emotional distress (i.e., expressions of anger, guilt, sadness, frustration, other), nonverbal emotional distress (i.e., crying, frowning, elevated tone of voice, other), or positive emotion (i.e., smiling, laughing, other). As for the physician’s response, we employed the VALUE approach [16] to identify and classify the empathetic statements made by the physician. The empathy shown by the physicians and the emotional cues of the patient and family members were assessed and recorded by handwritten notes during the conference by all joining medical team members. The research assistant, who is also a clinical psychologist, was responsible for the integration of every member assessment after the conference, and the draft of these observations was thoroughly reviewed by all healthcare professionals to reach a consensus. Identifiable information was removed after the verification of records by medical teams to ensure the anonymity and privacy of the participants. All analyses were conducted using SPSS version 23.0 (SPSS INC, Chicago, IL, USA).

### 2.4. Patient and Family-Reported Outcome toward the Smartphone-Enabled Telehealth Family Conference

At the end of the conference, family members were surveyed by an evaluation form that included seven questions aimed to understand the participants’ satisfaction and experiences towards the telehealth conferences. The evaluation form was based on the authors’ experiences conducting family conferences and previously validated questionnaires such as the quality of communication questionnaire from Curtis et al. after a careful literature review [39,40,41,42]. The questions were “How well did the medical team answer your questions about your loved one’s illness and treatment?”, “How well did the medical team listen to what you had to say?”, “How well did the medical team ask about the kinds of treatments your loved one would want if s/he could speak for him/herself?”, “How well did the medical team help your family decide about the treatments your loved one would want?”, “Overall, how would you rate the medical team’s communication with you during the conference?”, “How well did this conference help you understand the choices and decisions that may need to be made?”, and “Overall, how well did this conference meet your needs?”. The scoring system was a 10-point Likert scale, from ‘‘strongly disagree’’ [1] to ‘‘strongly agree’’ [10]. The Kuder–Richardson Formula 20, which is a measure of internal consistency reliability for measures with dichotomous choices, were used to assess this new measure. The coefficient showed 0.946 and indicated the measure to be a homogeneous test.

### 2.5. Patient Consent Statement and Ethical Approval

Patients and their family members had to provide consent for inclusion in the family conference and agree to answer the satisfaction survey questions. Written detailed records of the family conference were signed by all conference attendees and uploaded to the electronic medical record system of the hospital. The Research Ethics Committee of the National Taiwan University Hospital approved the study under the protocol 202004113RINC.

## 3. Results

### 3.1. Demographic Characteristics

During the COVID- 19 pandemic from February to May 2020, a total of 25 family conferences were conducted via telehealth technology. Among the 25 family conferences, 16 were conducted in the inpatient units, and six were conducted in the outpatient department. Demographic data regarding the patients and their designated family member participants were summarized in Table 1. The mean age of the patients was 72.9 ± 14.3 (mean ± SD), and the majority of them had an Eastern Cooperative Oncology Group (ECOG) score of 3–4. Nineteen (76%) were married, four (16%) were single, and two (8%) were widowed. Patients were presented in 16 (64%) of the conferences. On average, there were three family members participating in each telehealth family conference. Of the representative primary family caregivers, 11 (44%) were patients’ children, 7 (28%) were spouses, and 7 (28%) were other family members. The average length of the family conference was 31.9 ± 11.7 minutes. The first and the corresponding authors were the two main physicians conducting these 25 family conferences.

### 3.2. Patient and Family’s Emotional Cues (Verbal and Nonverbal)

The emotional cues as distress were observed in six (24%) patients nonverbally and five (20%) patients verbally. Among family members, nonverbal emotional distress was revealed in seven (28%) participants and five (20%) family members verbally. The sample quotes and behavior when revealing emotional distress by patient and family were demonstrated in Table 2. Verbal emotional distress included repeats of their concerns about the consequence of treatment withdrawal. The nonverbal expressions observed by medical teams included frowning, crying, speaking with shaking voices, fidgeting, and even sobbing. Nevertheless, apart from the negative emotions, we also recognized family members’ positive emotions, such as smiling and happily waving towards the camera at the end of the conference. Two (8%) patients and two (8%) family members were noticed having positive expressions.

### 3.3. Medical Team’s Emotional Work and VALUE Approach

**Figure 2:**
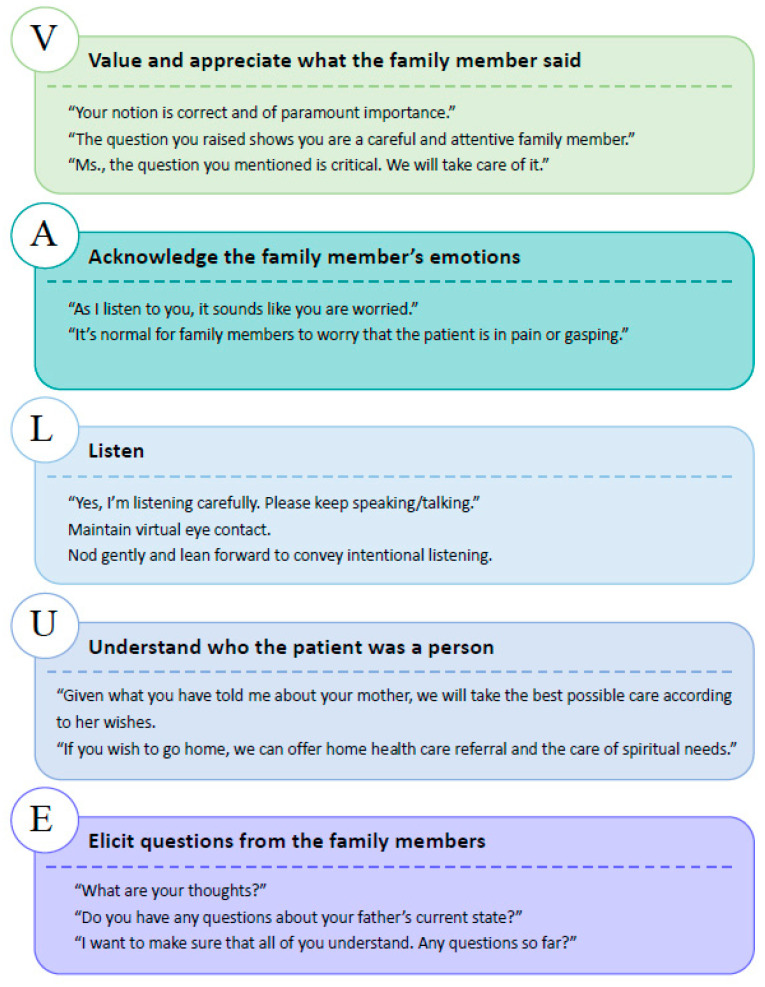
Physicians’ empathetic statements via VALUE approach.

In the telehealth family conferences, the medical team recognized patients’ and families’ emotional cues and made empathetic statements using the VALUE approach. The sample quotes and behaviors made by the physicians are demonstrated in Figure 2.

### 3.4. Patients’ and Family’s Reported Outcomes toward the Telehealth Family Conference

Patients and family members provided feedback at the end of the conference, and the outcome of communication in the telehealth family conference was summarized in Table 3. Based on the VALUE and PLACE approach, the reported outcome as satisfaction score toward the medical team’s overall communication during the conference was 8.7 ± 1.5 (mean ± SD). For the attitude on how well did the telehealth conference meet the family’s needs, the score was 9.0 ± 1.1.

## 4. Discussion

In this study, we demonstrated that by integrating the SDM model with the VALUE approach and PLACE emotional work, physicians could attend to the patient and family’s emotions with empathetic statements and connect to the participants through enhanced virtual presence. The communication experience of telehealth-based family conferences was very satisfying. Even though telehealth precludes the physical contact that is central to patient-centered care, explicit strategies can help physicians foster human connectedness with participants in virtual settings.

Emotional distress often emerges in the family conference when discussing difficult medical care options. As the observation of participants’ behavior in the study, expression of emotional distress by family members is not an uncommon occurrence, which was consistent with other groups’ findings [2,38]. Ideally, family conferences should provide a setting where distress and emotion can be openly discussed and safely supported by skillful and experienced physicians. Our family conference model suggests that empathetic communication can be mediated by using the VALUE approach and PLACE emotional work. Emotional work refers to physicians’ emotional engagement with the families and the content of the family conference [2]. While it was not possible to offer tissues or a comforting hand on the shoulder through telehealth, verbally acknowledging the emotion and expressing empathy (such as “It is normal for family members to worry that the patient is in pain or gasping.”) can be an effective substitute to ensure the family felt heard and their emotions were validated. A previous study shows that when physicians catch the emotion cue and respond appropriately, the conversation progresses and is deepened, which enables physicians to learn new information about the family’s fears and worries [43]. Empathy not only builds the physician–family relationship [44] but also helps physicians understand the patient and family’s values. Apart from emotional distress, we also found some family members express some positive emotions, such as smiling and happily waving towards the camera. This reflects that not only distressing emotions but also joys and comforts the family can experience in the telehealth family conference.

On the other hand, when it comes to telehealth communication, nonverbal communication is just as important as the words we use [17,45]. Gifford et al. developed 14 behavioral telehealth competencies for practicing, with one competency focusing on demonstrating appropriate “video presence” [46]. It includes ways to convey empathy across the screen by paying attention to the physician’s appearance on the screen, remaining visually attentive, and exaggerating facial expression, body language, and verbal responses at times [18,46]. In the study, the medical team used nonverbal gestures, such as nodding, leaning forward, and optimizing eye contact by looking directly at the camera to enhance the “video presence” and show signs of active listening. Liu et al. [47] suggested that physicians should exaggerate motions such as nods and large body actions in telehealth. Possessing appropriate verbal and nonverbal website manner skills is essential to conducting serious illness conversations during virtual context [36].

Family needs to feel cared for by their physician [48], and perceptions of physician empathy translate to perceptions of physician competence [49]. Our satisfaction results are in conjunction with recent researches, which found that video encounter is perceived to be a highly satisfactory approach to receive care [50,51,52]. Familys’ satisfaction with care conferences is higher with more patient/family-centered elements such as empathetic statements, question-asking, and acknowledgment of emotions [53,54,55]. Demiris et al. [56] suggested video-mediated encounters could engage in psychosocial issues and display empathy in telehealth sessions as face to face, resulting in lower patient anxiety and a higher level of satisfaction. It is encouraging that in our model, physicians can still apply their skills of listening, perceiving, and connecting via virtual platforms. As many countries have expanded the copayments and coverage of telehealth, our study provides insights into effective physician–patient virtual interaction, which is important for governments and stakeholders to facilitate telehealth family conferences necessitated by COVID-19 [57].

### Study Limitations

Several limitations should be considered in this study. First, this is a pilot study with a relatively small sample size. Further study on a larger scale is required to learn if the intervention is sufficiently robust to implement in various settings. Secondly, we did not videotape the conference. Some subtle behavioral interaction information might be missing. In future studies, video recording should be considered. Thirdly, we only had short-term patient and family reported outcomes as satisfaction analysis. The willingness to participate in our virtual family conferences may add bias to the result of satisfaction surveys, which may lead to an overestimation of the level of satisfaction [58]. To obtain a fuller understanding of the implementation of telehealth with the VALUE approach and PLACE emotional work, the long-term impact on patient clinical management and family bereavement requires follow-up investigation. Lastly, the study lacked face-to-face family conferences as a comparison because of the strict visitor restriction during the COVID-19 pandemic. Future studies assessing the effectiveness of the model in comparison with face-to-face contact are warranted.

## 5. Conclusions

It is of paramount importance to maintain person-centered care under the tremendous impacts of global COVID-19 pandemics. The modern technology of telehealth offered the opportunities to deliver good doctor–patient communication even under the strict infection control measures as restrictions of face-to-face interviews. However, the anxiety of the general public under threats of the pandemic and the burnout of medical professionals in the health care system may manifest as emotional exhaustion and disengagement during clinical encounters. Our study demonstrated that the telehealth family conference based on the 3-talk SDM model with the integration of the VALUE approach and PLACE emotional work is satisfying to the patients and family. With the help of the VALUE approach, we can better understand the patient as a person and elicit questions from the family members. Understanding the patient and family’s values allowed physicians to provide care that was aligned with the patient’s preference and thereby build a trusting relationship [59]. Communication skills that are applicable to face-to-face can be applied to telehealth as well. The model offered practical guidance for health care professionals in virtual family conferences to establish sound communication and rapport. The model could be adopted worldwide to improve the quality of care when using telehealth, not only during the COVID-19 pandemic but also for future care. Furthermore, the model had implications for the governments to adopt the policy on encouraging telehealth for medical care, and the evidence might help stakeholders to broaden the reimbursement of the payment for telehealth in the medical insurance system.

## Figures and Tables

**Table 1:** Demographic characteristics of patients and family participants in the telehealth-based family conferences.

Characteristic	No. (%)
**Patient’s baseline characteristics**	
Age, (mean ± SD)	72.9 ± 14.3
Marital status	
Married	19 (76%)
Single	4 (16%)
Widowed	2 (8%)
Education	
Illiterate	2 (8%)
Elementary school	7 (28%)
Junior high school	5 (20%)
High school	4 (16%)
Bachelor	6 (24%)
Master or PhD	1 (4%)
ECOG	
1	0 (0%)
2	1 (4%)
3	9 (36%)
4	15 (60%)
Whether the patient participates in family conference	
Yes	16 (64%)
No	9 (36%)
**Family participants’ characteristics**	
The number of participants (mean ± SD)	3.52 ± 1.42
The representative primary family caregiver’s relationship to the patient	
Parents	1 (4%)
Spouse	7 (28%)
Daughter/Son	11 (44%)
Sibling	3 (12%)
Others	3 (12%)

ECOG: Eastern Cooperative Oncology Group, SD: standard deviation.

**Table 2:** Patients’ and family’s emotional cues.

Category of Emotional Cue	Patient	Family Members
Nonverbal emotional distress Observed, *N* (%) Sample behaviors	6 (24%) Frowning Crying Shaking voices	7 (28%) Sobbing Fidgeting
Verbal emotional distress Observed, *N* (%) Sample quotes	5 (20%) Worried about being a burden to his wife. Do not want to receive any treatment (including hospice treatment). Do not want to go back home.	5 (20%) Worried about withdrawal of treatment. Ask questions about withdraw of treatment repeatedly. Can we deal with unexpected situations?
Positive emotion expression Observed, *N* (%) Sample behaviors	2 (8%) Smiling	2 (8%) Smiling Happily waving towards the camera

**Table 3:** Patients and family’s reported outcomes toward the telehealth family conferences.

Evaluation Questions	Mean (SD)
1. How well did the medical team answer your questions about your loved one’s illness and treatment?	8.7 (0.9)
2. How well did the medical team listen to what you had to say?	8.7 (0.9)
3. How well did the medical team ask about the kinds of treatments your loved one would want if s/he could speak for him/herself?	8.9 (0.9)
4. How well did the medical team help your family decide about the treatments your loved one would want?	8.9 (1.1)
5. Overall, how would you rate the medical team’s communication with you during the conference?	8.7 (1.5)
6. How well did this conference help you understand the choices and decisions that may need to be made?	9.0 (0.9)
7. Overall, how well did this conference meet your needs?	9.0 (1.1)

SD: standard deviation.

## Data Availability

The data presented in this study are available upon reasonable request from the corresponding author.
